# The design of an “H” joystick for closed reduction and its application in segmental and comminuted femoral shaft fractures: an innovative technique

**DOI:** 10.1186/s13018-020-01898-x

**Published:** 2020-08-26

**Authors:** Zhaofeng Jia, Shijin Wang, Tinghui Xiao, Wei Jiang, Tianjian Zhou, Qisong Liu, Guangheng Li, Xinjia Hu

**Affiliations:** 1 Department of Osteoarthropathy, Shenzhen People’s Hospital, The Second Clinical Medical College of Jinan University and the First Affiliated Hospital of Southern University of Science and Technology, Shenzhen, 518035 Guangdong Province China; 2 Department of Orthopaedics, Taian City Central Hospital, Taian City, 271000 Shan dong Province China; 3 Institute for Regenerative Medicine, Texas A&M Health Science Center College of Medicine, Temple, TX 76502 USA

**Keywords:** “H” joystick, Femoral shaft fracture, Closed reduction, Locked intramedullary nailing

## Abstract

**Background:**

Closed reduction and locked intramedullary nailing has become a common surgical method in the treatment of femoral shaft fractures. Overlap and rotation displacements can usually be corrected through the use of an orthopedic traction table. However, lateral displacement and angulation persist.

**Methods:**

In this paper, we describe a joystick that can be used in the closed reduction of a fracture. It can correct lateral displacement and angulation, and has the advantage of multi-direction reduction. The device described in this paper includes two parallel horizontal joysticks, one vertical main joystick and four assistant rods. Moreover, there are many specific spacing holes in the two parallel horizontal joysticks and a groove structure in the vertical main joystick. When the main “H” joystick is pressed, it can adjust lateral displacements and angulation because of the lever principle. The distance between parallel horizontal joysticks and assistant rods can be adjusted to the fracture position and body mass index of different patients.

**Results:**

The study participants consisted of 11 males and 5 females with a mean age of 31.0 years. All participants had good closed reduction and achieved bony union without any complications such as infection, nerve injury, non-union, malunion, and limb length discrepancy. By using an “H” joystick, closed femoral shaft fracture reduction and locked intramedullary nailing becomes simpler and faster.

**Conclusion:**

Based on the use of this instrument, we can easily and conveniently obtain the correct reduction situation, which leads to better surgical results. This device can be applied in the reduction of clinical femoral fractures and gradually extended to the reduction of other fractures.

## Introduction

In clinical medical treatment, it is common for adults who sustain high-energy trauma to suffer from femur, tibia and humerus shaft fractures [1, 2]. Closed reduction and locked intramedullary nailing is generally accepted as a standard treatment for femoral shaft fractures because of its satisfactory union rate in a stable biomechanical environment [3–5]. During femur shaft fracture operations, through traction using an orthopedic traction table, displacement of overlap, and rotation can be basically corrected. However, lateral displacement and angulation persist [6]. The conventional method is for doctors and assistants to reduce the displacement by hand, which is clearly time-consuming and laborious. Moreover, the displacement accuracy is poor, and it requires greater exposure to X-rays, which cause unnecessary damage to both medical personnel and patients [7–10]. Therefore, it is important and urgent for doctors to invent a type of device that can realize the effective reduction of femoral shaft fractures. In this regard, we designed the “H” joystick, applied it in femoral shaft fracture operations and achieved satisfactory results.

## Materials and methods

### Ethics statement

This study was approved by the Ethics Committee of Shenzhen People’s Hospital at Jinan University. All volunteers gave informed consent prior to participating in the study.

### Characteristics and application principles of the “H” joystick device

Locked intramedullary nailing for the treatment of femoral shaft fractures requires careful preoperative planning and the application of multiple techniques to achieve suitable fracture reduction [11–13]. We will describe the details of the device we designed and the method of application. Moreover, we have applied for a patient.

The theoretical basis of the design is the leverage principle, which is used to reduce fractures by applying pressure on the skin without injury [14]. The device in this report includes two parallel horizontal joysticks, one vertical main joystick and four assistant rods. Moreover, there are many specific spacing holes in two parallel horizontal joysticks and a groove structure in vertical main joystick. By pressing the main “H” joystick, lateral displacement and angulation can be adjusted because of the lever principle. The distance between parallel horizontal joysticks and assistant rods can adjust to the fracture position and body mass index of different patients.

Another important consideration is the material used to construct the device. The material we chose is invisible to X-rays and works in high-temperature environments. It does not contain any bolts or other metal components. It has the features of high intensity and low density. Therefore, when medical personnel make X-ray examinations for fracture reduction, it does not affect the physician’s judgment of the reduction, thereby improving the efficiency of the reduction procedure, relieving patient pain and reducing patient X-ray exposure time. Finally, the device can be disinfected with high temperatures and used in a sterile environment.

**Fig. 1:**
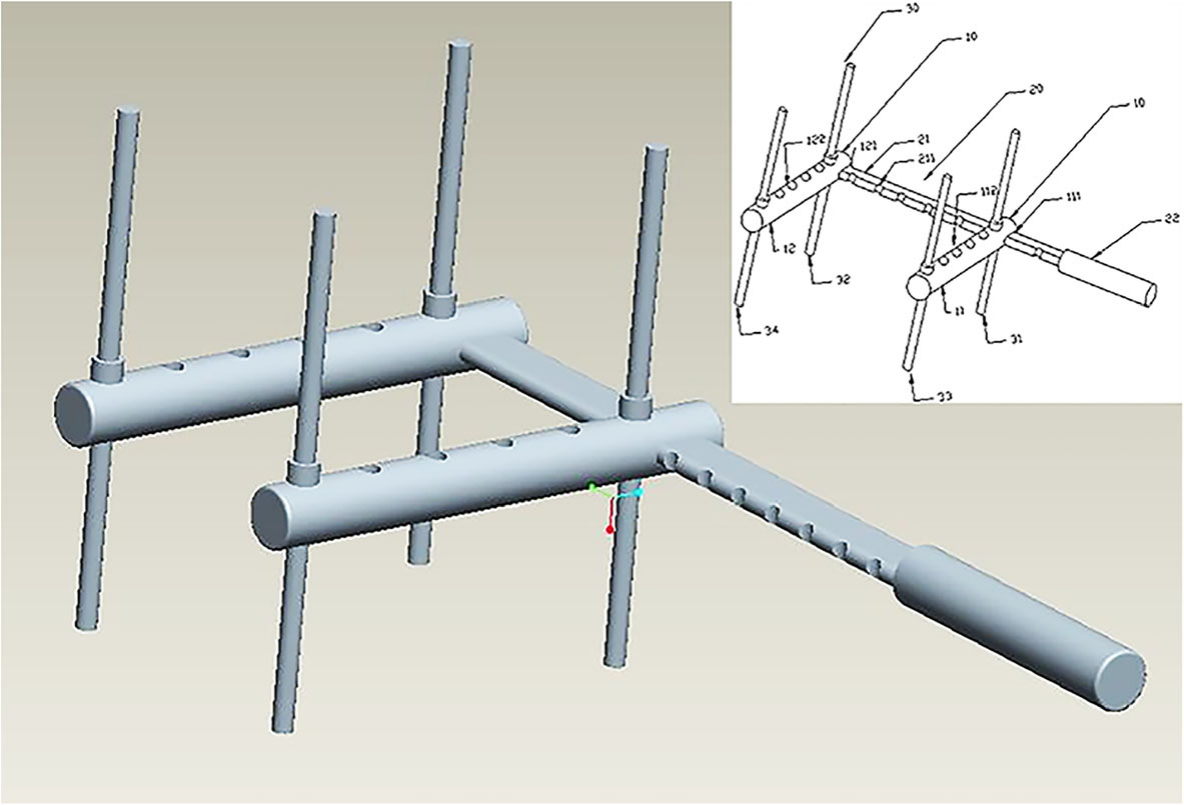
The component and assembly relationship of the “H” joystick

**Fig. 2:**
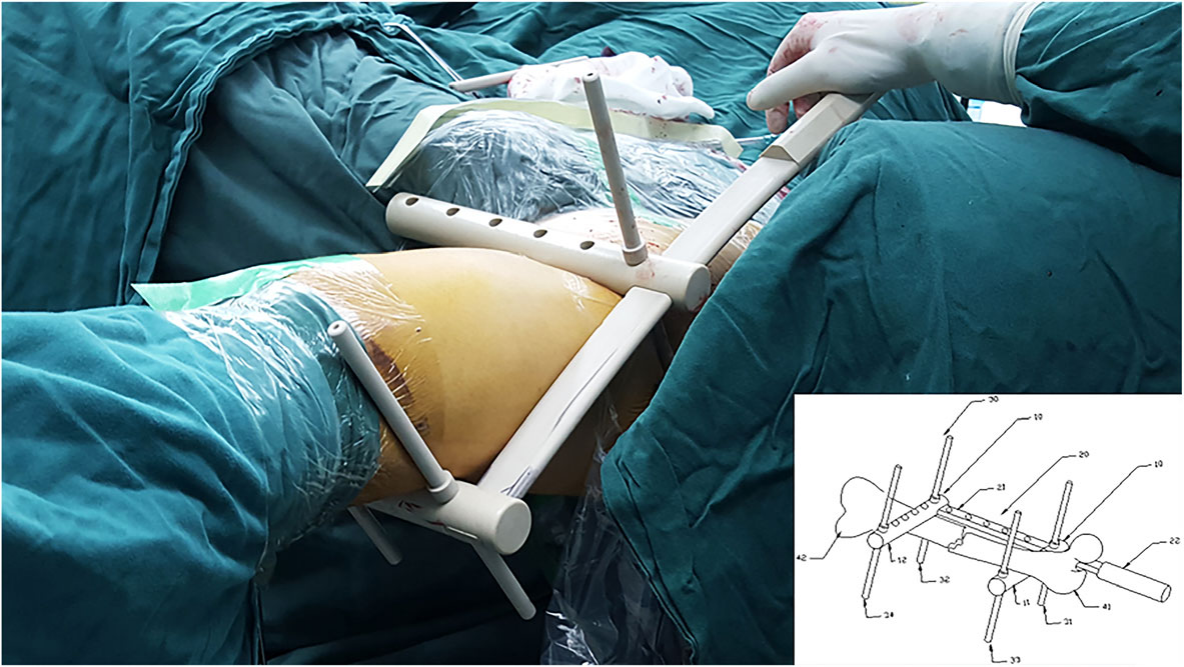
Fracture reduction assisted by the “H” joystick

The main operation steps are described as below:

1. Assemble the device. Connect the main joystick with the two parallel horizontal joysticks and four assistant rods, as shown in Fig. 1 and Figure S1.

2. Place assembled joysticks on the fracture sites and adjust the lateral displacement or angulation to reduce the fracture.
3. When the fracture reduction is satisfactory by X-ray, insert the “gold finger” along the medullary canal, going through the fractured face smoothly, and then penetrate the guide wire. Another method is to insert the intramedullary nail from the opening directly and complete the operation as shown in Fig. 2

We can adopt different devices using methods according to different fracture types. This report will describe operation skills in detail for both simple and complex fractures.

If the fracture type is an up-and-down or lateral displacement, using parallel joysticks 10, the main joystick 20 and two assistant rods 30 can reduce the fracture. For example, assume that the femoral shaft fracture type of the patient is that broken bone 41 is downwards and broken bone 42 is upwards, as shown in Fig. 2. Insert the main joystick 20 into the two parallel horizontal joysticks’ homolateral first holes 111 and 121 successively. According to the length and diameter of the fracture site, choose the suitable groove 211 in the main joystick 20. After insuring that the second holes 112 and 122 in the parallel horizontal joysticks align at the groove 211 lengthways, insert the first assistant rod 31 and the second assistant rod 32 and fix all the parallel horizontal joysticks and assistant rods. Next, insert the assembled device into the fracture site and make the first parallel joystick 11 below the broken bone 41 and the second parallel joystick 12 below the broken bone 42. Under X-ray, the physician holds the handle 21 and moves the main joystick 20 according to the relative position of the fracture. Because of the lever principle, the first parallel joystick 11 will support the broken bone 41 upwards, and the second parallel joystick 12 will press the broken bone 42 downwards. Therefore, we can reduce the fracture of bones 41 and 42. Similarly, other unidirectional fractures can be reduced. It is notable that the entire operation is simple and time-saving and that only one operating physician is required to complete the reduction.

If the fracture type is simultaneously multi-directional and complex, physicians need to use two more assistant rods to complete the reduction. In this case, the femoral shaft fracture type in these patients is that broken bone 41 is oriented downwards and broken bone 42 is oriented upwards and rightwards. On the basis of the previous example, insert the third assistant rod 33 and the fourth assistant rod 34 into the suitable holes in the parallel horizontal joysticks. The assistant rods 31 and 32 play a part in fixation and support. In this orientation, the third assistant rod 33 could press the broken bone 41 rightwards, and the fourth assistant rod 34 could press the broken bone 42 leftwards. Therefore, physicians can use this device flexibly to adapt different situations.

We performed a retrospective analysis of the medical records of patients treated for all femoral shaft fractures between 1 February 2013 and 30 May 2016 at our institution. During this period, there were 51 femoral shaft fractures. Patients with open fractures, pathological fractures, metabolic bone disease, or neuromuscular disorders were excluded from our analysis. We selected 16 patients who were treated with an “H” joystick as objects of study. We processed all 16 patients’ radiographs and medical data statistically, including age, height and weight, sex, side, mechanism injury type, fracture location, and fracture type classified by AO (Arbeitsgemeinschaft für Osteosynthesefragen) system [15]. The study was performed according to the guidelines stated in the Declaration of Helsinki [16], and the study protocol was approved by the local ethics committee.

All 16 patients, including 11 male patients (68.8%) and 5 female patients (31.2%), had a unilateral femoral shaft fracture. The mean age of patients was 31.0 years old (ranged from 20 to 55 years old). The weight of patients was an average of 63.3 kg (range from 49 to 78 kg). There were 6 cases (37.5%) injured on the right and 10 cases (62.5%) injured on the left. The injury types include 7 cases (43.8%) of traffic accidents, 5 cases (31.2%) of falls, and 4 cases (25%) of sports injuries. The fracture location was divided to the proximal area, the proximal area, and the distal area, with 6 cases (37.5%), 8 cases (50%), and 2 cases (12.5%), respectively. There were nine cases (56%) of AO fracture type A, five cases (31%) of AO fracture type B, and two cases (13%) of AO fracture type C. Moreover, there were 4 cases of simultaneous combined injury and 2 cases of anamnesis such as hypertension and diabetes. The details are shown in Table 1.

**Table 1.** Patient characteristics

No.	Age (years.)	Height (cm)/weight (kg)	Sex	Side	Mechanism injury type	Fracture location	Fracture type (AO)
1	23	163/49	F	L	Traffic accident	Mid 1/3	A3
2	42	175/63	M	L	Sports	Mid 1/3	A2
3^b^	38	155/54	F	L	Traffic accident	Dis 1/3	A2
4	20	178/67	M	R	Traffic accident	Mid 1/3	B3
5^b^	40	154/45	F	L	Sports	Proximal 1/3	A3
6^b^	55	180/78	M	L	Traffic accident	Proximal 1/3	C3
7	31	176/66	M	R	Sports	Mid 1/3	B1
8^a^	28	165/62	M	L	Fall	Mid 1/3	A3
9	32	157/63	F	R	Traffic accident	Proximal 1/3	A3
10	34	177/78	M	L	Fall	Mid 1/3	C2
11	29	163/56	M	R	Traffic accident	Proximal 1/3	A1
12^a^	28	175/77	M	L	Traffic accident	Proximal 1/3	B2
13	42	176/71	M	L	Sports	Mid 1/3	A3
14	36	162/52	F	R	Fall	Proximal 1/3	B1
15^a^	25	169/63	M	L	Fall	Dis 1/3	A3
16	22	177/69	M	R	Fall	Mid 1/3	B2

^a^Patients suffering from anamnesis such as hypertension or diabetes

^b^Patients suffering from simultaneous combined injury such as skull fracture, pulmonary contusion, or upper limb fracture

### Statistical analysis

All data were analyzed using SPSS (Statistical Product and Service Solutions) 18.0 statistical software in this study. The measurements were expressed as mean ± standard deviation (*x* ± *s*). Comparisons between groups were performed using the one-way ANOVA followed by *t* tests, and data counting was analyzed using *χ*^*2*^ tests. *P* values less than 0.05 were considered as statistically significant different.

## Results

As mentioned previously, we treated 16 patients suffering from femoral shaft fractures with an “H” joystick. During the operation of reduction and locked intramedullary nailing, all 16 patients received closed reduction. The mean operation time was 85 min (ranging from 40 to 105 min), which is less than the same operation without using an “H” joystick. Because the device is non-invasive, surgical tresis and blood loss were reduced in all operations.

After treating all 16 patients with an “H” joystick, we performed a longitudinal study and observed their recovery. The follow-up survey lasted 13 months (range from 6 to 18 months) on average. All patients achieved bone union. The duration until union averaged 6 months (range from 5 to 13 months). There were no complications such as infection, nerve injury, nonunion, malunion, or limb length discrepancy after operation, and there were no hip and knee joint motion limitations, pain, limping, or gait abnormalities.

**Fig. 3:**
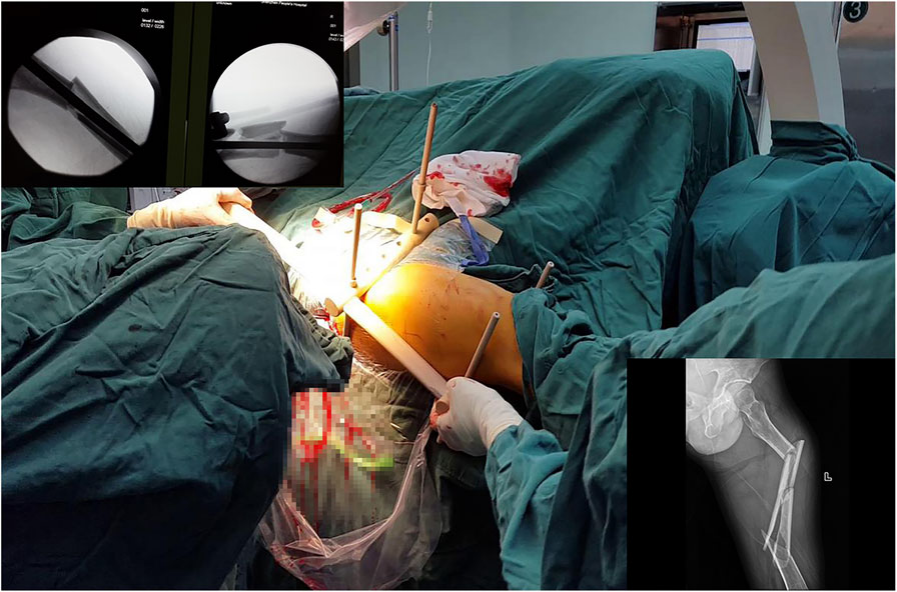
A 34-year-old man who sustained a left femoral shaft fracture is undergoing closed reduction by an “H” joystick

**Fig. 4:**
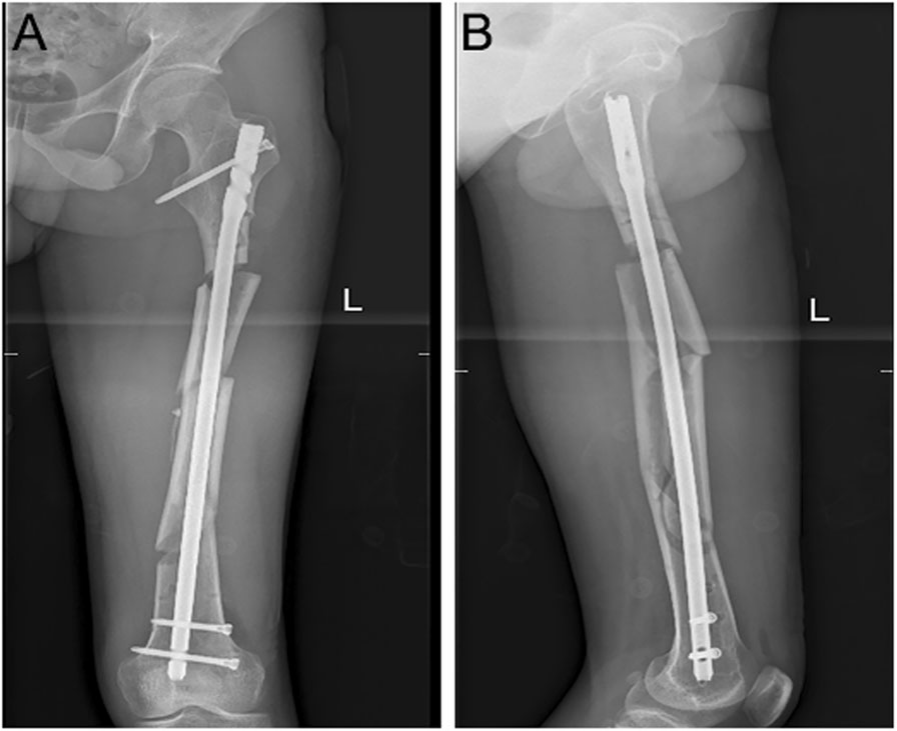
A 34-year-old man who sustained a left femoral shaft fracture after closed intramedullary nailing. **a** Anteroposterior view. **b** Lateral view

**Fig. 5:**
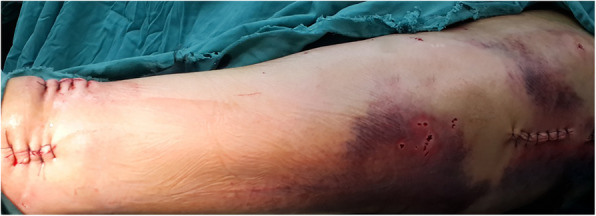
A 34-year-old man who sustained a left femoral shaft fracture after closed intramedullary nailing

**Fig. 6:**
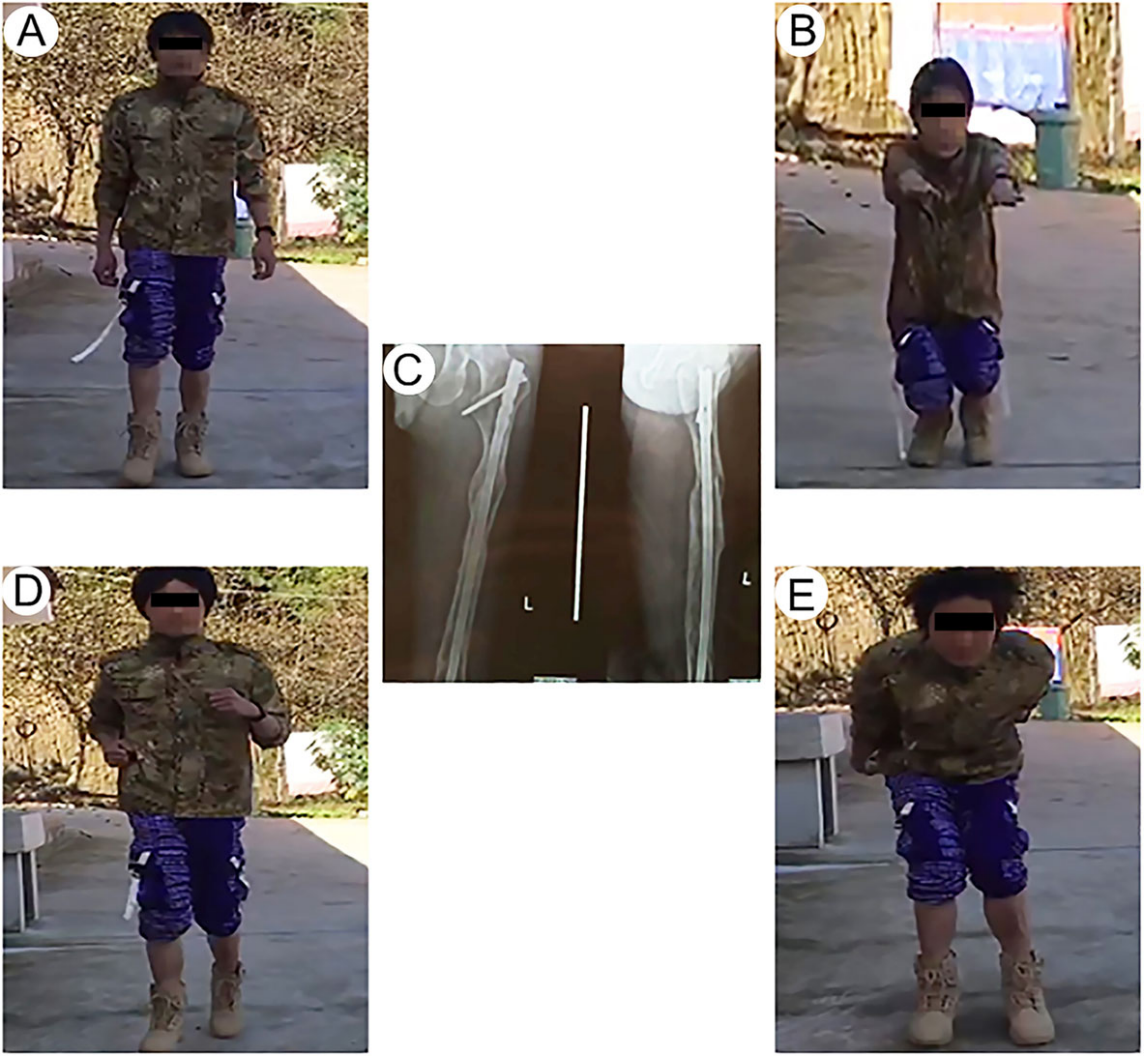
X-ray radiograph showing the fracture after 12 months with complete callus bridging the fracture (**c**) and full functional range of motion when walking (**a**), squatting (**b**), running (**d**), jumping (**e**) with full weight-bearing

Particularly, one of the patients suffered from femoral shaft fracture with multi-segments. With the help of Kirschner wire and blocking screws [17], the fracture realized closed reduction. The patient was 34 years old and male, and the fracture was on his left femur. We performed a closed reduction and locked intramedullary nailing with an “H” joystick, as shown in Fig. 3. We clearly observed good closed reduction (Fig. 4). After implanting the intramedullary nail, the surgical tresis was small (Fig. 5). X-ray radiograph showing the fracture after 12 months with complete callus bridging the fracture and full functional range of motion with full weight-bearing (Fig. 6).

## Discussion

The closed intramedullary nailing technique is the gold standard treatment for femoral shaft fractures [3, 11, 18]. A key procedure of the intramedullary nailing technique is to insert the guide wire into the desired position from the proximal medullary cavity into the distal cavity [19–21]. However, residual displacement after the initial closed reduction of the femoral shaft fracture may make insertion of the guide wire and subsequent nail fixation difficult [22–25].

In the present study, we invented an “H” joystick to facilitate closed reduction non-invasively in the operation of locked intramedullary nailing. With the help of the “H” joystick, medical personnel can easily reduce lateral displacement or angulation and improve reduction accuracy. Moreover, the device has many other advantages such as its non-invasive nature, capacity for multi-direction reduction, good reliability, broad applicability, and easy disassembly. These strengths help physicians complete surgeries more proficiently and conveniently.

It is well understood that the underlying structure and material have a great effect on the functions and features of a device. The “H” joystick uses an elegant and simple structure design, consisting of only 7 joysticks. Because of its structure, a physician can acclimate to it quickly and operate it skillfully. It is disassembled easily and the reliability of the device can be increased. Additionally, the “H” joystick is made of the material that can be penetrated by X-rays and that resists high temperatures, which ensures X-ray images are not affected and the structure is not deformed by high temperatures. Different components of the “H” joystick can be used to close both simple and complex fractures. The “H” joystick is non-invasive, which means smaller operation tresis and less blood loss than the device being put into the body. As given data previously, the use of the device decreases the operation time and eases patient pain. According to the results of follow-up survey after operation, all patients achieved bone union, and there were no complications.

In addition, the “H” joystick can not only be used in intramedullary nail fixation for assisting reduction but also plays an important role in closed pre-reduction and making plaster or splint external fixations. The assistant draws the distal limb, and then the operator reduces the fracture with the “H” joystick according to the aforementioned method. At this time, the joystick does not need to be sterilized and can be assembled and disassembled at any time.

The “H” joystick has several limitations. First of all, this instrument can only be used with an orthopedic traction bed. Second, the joystick cannot fully solve the problem of rotation displacement. Last, it is not accurate enough for the fracture reduction, and needs to be improved.

## Conclusion

The “H” joystick has solved the problem of the closed reduction of fractures in the clinic and achieved good clinical results in femoral shaft fractures. It can be widely used for reduction of femoral shaft fractures and gradually extended to other fractures. However, there are some limitations. In surgeries, once the closed reduction occurs, we perform the subsequent steps. Therefore, it is impossible and unethical to take a comparative path and assessment of one patient who was treated with and without the “H” joystick. Therefore, we did not have a comparison group. More importantly, the mean follow-up period was too short to evaluate the results thoroughly.

## Supplementary information

**Additional file 1: Figure S1.** The sketch of the “H” joystick

## Data Availability

All the data and materials can be found in the manuscript.

## Abbreviations

AO: Arbeitsgemeinschaft für Osteosynthesefragen
SPSS: Statistical Product and Service Solutions
