# Supplementary figures and images for: The design of an “H” joystick for closed reduction and its application in segmental and comminuted femoral shaft fractures: an innovative technique
Source: J Orthop Surg Res. 2020 Aug 26;15:357. doi: 10.1186/s13018-020-01898-x (PMC7449011; doi:10.1186/s13018-020-01898-x)

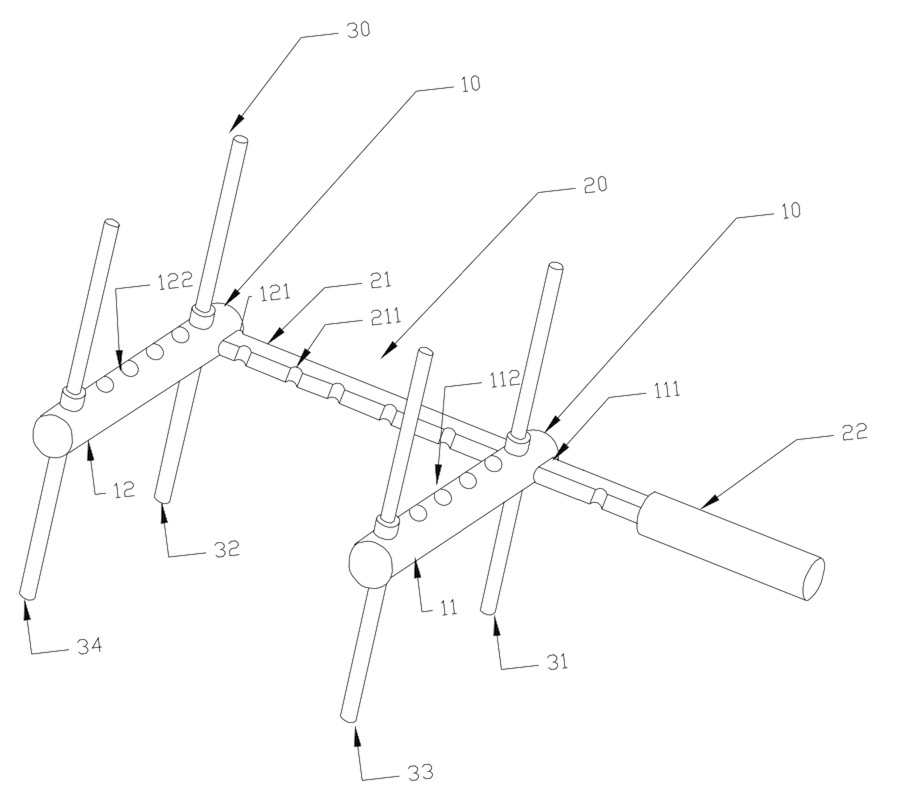

Supplement: Supplementary file 1 — Additional file 1: Figure S1. The sketch of the “H” joystick [file 13018_2020_1898_MOESM1_ESM.tiff]
